# Evaluating the use of oral pre-exposure prophylaxis among pregnant and postpartum adolescent girls and young women in Cape Town, South Africa

**DOI:** 10.3389/frph.2023.1224474

**Published:** 2023-09-19

**Authors:** Nehaa Khadka, Pamina M. Gorbach, Dorothy C. Nyemba, Rufaro Mvududu, Nyiko Mashele, Marjan Javanbakht, Roch A. Nianogo, Grace M. Aldrovandi, Linda-Gail Bekker, Thomas J. Coates, Landon Myer, Dvora L. Joseph Davey

**Affiliations:** ^1^ Department of Epidemiology, Fielding School of Public Health, University of California Los Angeles, Los Angeles, CA, United States; ^2^ Division of Epidemiology and Biostatistics, School of Public Health and Family Medicine, University of Cape Town, Cape Town, South Africa; ^3^ Wits RHI, University of the Witwatersrand, Johannesburg, South Africa; ^4^ David Geffen School of Medicine, University of California Los Angeles, Los Angeles, CA, United States; ^5^ The Desmond Tutu Health Foundation, University of Cape Town, Cape Town, South Africa

**Keywords:** South Africa, AGYW, adherence, breastfeeding, cohort studies, oral pre-exposure prophylaxis, pregnant

## Abstract

**Background:**

Adolescent girls and young women (AGYW) in South Africa are at a higher risk of acquiring HIV. Despite the increasing availability of daily oral pre-exposure prophylaxis (PrEP) for HIV prevention, knowledge on PrEP use during pregnancy and postpartum periods at antenatal care (ANC) facilities remains inadequate.

**Methods:**

Data from HIV-uninfected pregnant women in Cape Town, South Africa, were used in this study. These women aged 16–24 years were enrolled in the PrEP in pregnancy and postpartum (PrEP-PP) cohort study during their first ANC visit. Using the PrEP cascade framework, the outcomes of the study were PrEP initiation (prescribed tenofovir disoproxil fumarate and emtricitabine at baseline), continuation (returned for prescription), and persistence [quantifiable tenofovir diphosphate (TFV-DP) in dried blood samples]. The two primary exposures of this study were risk perception for HIV and baseline HIV risk score (0–5), which comprised condomless sex, more than one sexual partner, partner living with HIV or with unknown serostatus, laboratory-confirmed sexually transmitted infections (STIs), and hazardous alcohol use before pregnancy (Alcohol Use Disorders Identification Test for Consumption score ≥ 3). Logistic regression was used to examine the association between HIV risk and PrEP, adjusting for *a priori* confounders.

**Results:**

A total of 486 pregnant women were included in the study, of which 16% were “adolescents” (aged 16–18 years) and 84% were “young women” (aged 19–24 years). The adolescents initiated ANC later than the young women [median = 28 weeks (20–34) vs. 23 weeks (16–34), *p* = 0.04]. Approximately 41% of the AGYW were diagnosed with sexually transmitted infection at baseline. Overall, 83% of the AGYW initiated PrEP use during their first ANC. The percentage of PrEP continuation was 63% at 1 month, 54% at 3 months, and 39% at 6 months. Approximately 27% consistently continued PrEP use through 6 months, while 6% stopped and restarted on PrEP use at 6 months. With a higher risk score of HIV (≥2 vs. ≤1), the AGYW showed higher odds of PrEP continuation [adjusted odds ratio: 1.85 (95% CI: 1.12–3.03)] through 6 months, adjusting for potential confounders. Undergoing the postpartum period (vs. pregnant) and having lower sexual risk factors were found to be the barriers to PrEP continuation. TFV-DP concentration levels were detected among 49% of the AGYW, and 6% of these women had daily adherence to PrEP at 3 months.

**Conclusions:**

AGYW were found to have high oral PrEP initiation, but just over one-third of these women continued PrEP use through 6 months. Pregnant AGYW who had a higher risk of acquiring HIV (due to condomless sex, frequent sex, and STIs) were more likely to continue on PrEP use through the postpartum period. Pregnant and postpartum AGYW require counseling and other types of support, such as community delivery and peer support to improve their effective PrEP use through the postpartum period.

**Clinical Trial Number:**

ClinicalTrials.gov, NCT03826199.

## Introduction

Adolescent girls and young women (AGYW, aged 16–24 years) in South Africa have a higher risk of acquiring HIV. The Joint United Nations Programme on HIV/AIDS (UNAIDS) reported that in 2021, approximately 250,000 AGYW were infected with HIV worldwide and six out of seven cases of HIV infections among adolescents (aged 15–19 years) in sub-Saharan Africa occurred among girls (1). Despite representing only 10% of the total population in sub-Saharan Africa, AGYW accounted for 25% of all acute HIV infections (2). AGYW are at a higher risk of acquiring HIV (3), and they may acquire HIV 5–7 years earlier than their male peers (3, 4). Thus, UNAIDS aims to reduce new cases of HIV infections among AGYW to less than 50,000 cases by 2025 (1).

Acquiring HIV is especially high during pregnancy and postpartum periods. AGYW have an immature cervix that has greater proportion of an exposed genital mucosa susceptible to HIV, and they also have higher levels of genital inflammation and hormonal effects compared with older women (5). The factors associated with higher risk of acquiring HIV among AGYW include age-disparate sexual partners, multiple partners, unknown serostatus of the partner, low marital or cohabitation prevalence rates, earlier sexual debut, gender-based violence, lack of sexual education, frequent condomless sex, and sexually transmitted infections (STIs) (6–8). In South Africa, the prevalence rate of pregnancy in adolescents (aged <19) is estimated at 20% (9), and 76% of these pregnancies are unintended (10). Young women without the intention of getting pregnant usually delay seeking antenatal care (ANC), and associated HIV testing and care, compared with those with planned pregnancies. Moreover, the risk of vertical transmission is much higher among those with HIV infections during pregnancy/postpartum than that of those who are already living with HIV (11). In 2021, 22,000 cases of HIV infections occurred during pregnancy or breastfeeding periods in Eastern and Southern Africa (1). The pooled HIV incidence rate during pregnancy and postpartum periods was found to be 3.6 per 100 person-years (95% CI: 1.2–11.1) in sub-Saharan Africa (12), which met the UNAIDS threshold for substantial risk of acquiring HIV (1). Therefore, the prevention of acquiring HIV throughout pregnancy and postpartum periods is particularly not only important for maternal health but also pivotal in eliminating vertical HIV transmission (11).

The South African National Department of Health supports the provision of oral pre-exposure prophylaxis (PrEP) and HIV prevention counseling as part of a comprehensive combination prevention strategy for AGYW and pregnant and breastfeeding women who are at substantial risk of acquiring HIV (1, 13). Oral PrEP with tenofovir disoproxil fumarate and emtricitabine (TDF-FTC) is an antiretroviral medication that can be taken daily by HIV-negative individuals before HIV exposure to prevent acquiring the infection; however, high adherence to this medication during periods of high HIV risk is required for PrEP to be effective (11). The PrEP cascade, an analogous extension of the HIV care cascade (14), provides a quantifiable framework for measuring the progress of HIV prevention methods and PrEP delivery. It illustrates the following stages of PrEP delivery: PrEP eligibility, initiation, persistence on PrEP during periods of high HIV risk, and adherence to PrEP for sufficient protection from HIV (15). Moreover, prior studies have reported that PrEP delivery for AGYW poses unique challenges, such as pill burden and stigma from taking an oral PrEP and for being pregnant (16, 17). Studies on pregnant and postpartum women have also identified delivery patterns that were unique to pregnancy, such as the high attrition rates during postpartum periods (18–22). However, there is a gap in knowledge for PrEP cascade and adherence studies among AGYW during pregnancy and postpartum periods.

We utilized the PrEP cascade among pregnant AGYW to examine PrEP initiation, continuation through 6 months, and persistence at a busy ANC facility in Cape Town, South Africa. We also evaluated the association between baseline HIV risk and PrEP delivery outcomes to inform the national and regional PrEP programs that are scaled up for pregnant/postpartum women and AGYW.

## Methods

### Study population

We used data from the PrEP in Pregnancy and Postpartum (PrEP-PP) study, a prospective cohort of 1,200 women based in Cape Town, South Africa, to evaluate PrEP initiation, continuation, and persistence among a subset of pregnant and postpartum AGYW. The study's methodology has been described in detail in another study (21). In summary, PrEP-PP study participants (aged ≥16 years) were recruited into the study during their ANC visit at a public health clinic from August 2019 to October 2021 and were followed up through 12 months postpartum. Interested study participants provided written informed consent in English or their local language (isiXhosa). The participants were eligible for the study if they were confirmed to be pregnant, not living with HIV (confirmed by a fourth-generation rapid HIV antigen/antibody test from Abbott Laboratories), and negative for hepatitis B surface antigen (confirmed by a rapid hepatitis B surface antigen test from Abbott Laboratories).

### Enrollment and measurements

Upon enrollment, the study staff administered a baseline survey collecting participant's demographic information, clinical characteristics, and behavioral HIV risk factors using REDCap, a secure web-based application. The participants underwent a point-of-care testing for STIs, and those participants diagnosed with sexually transmitted infection (STI) were provided with treatment according to the South African national guidelines for STI (23). Pregnant AGYW underwent HIV testing and counseling, with an offer to start using PrEP as part of a comprehensive combination prevention strategy along with promoting condom use and HIV prevention counseling, regardless of the responses to behavioral HIV risk factors and STI status. The study participants interested to start using PrEP had their blood tested to confirm whether their baseline creatinine levels (i.e., glomerular filtration rate of >60) met the clinical eligibility for PrEP or not. The participants who started using PrEP were provided with a 1-month supply of Truvada (TDF-FTC or “PrEP”).

Follow-up visits were conducted at 1, 3, and 6 months and were scheduled with the participants’ regular ANC visits until delivery. At the 1-month visit, the participants were provided with a PrEP refill. At the 3- and 6-month visits, the participants completed brief follow-up surveys regardless of PrEP use through interviews conducted by trained study staff in a private clinic room. Furthermore, the participants were supplied with additional PrEP prescriptions (for those interested); dried blood spot (DBS) samples were also collected from those who reported taking PrEP in the last 30 days during follow-up.

### Ethics

The study was approved by the Human Research Ethics Committee at the University of Cape Town (#297/2018) and by the Institutional Review Board of the University of California, Los Angeles (IRB#18-001622). This study followed the reporting guidelines based on the Strengthening the Reporting of Observational Studies in Epidemiology (STROBE).

### Outcomes: PrEP initiation, continuation, and objective persistence

We evaluated PrEP initiation, continuation (1, 3, and 6 months), consistent continuation through 6 months, and objective persistence (3 and 6 months). PrEP initiation was defined as accepting and receiving a PrEP prescription at the baseline visit, which was also their first ANC visit. PrEP continuation was defined as receiving a PrEP prescription at each study visit after the baseline visit among those who initiated PrEP at baseline. PrEP continuation through 6 months was defined as attending the study visits and receiving a PrEP prescription at all study visits (1, 3, and 6 months) among those who initiated PrEP at baseline compared with those who did not attend the study visits or those who reported discontinued PrEP use.

Objective PrEP persistence was measured using erythrocyte intracellular tenofovir diphosphate (TFV-DP) concentration levels detected by liquid chromatography and mass spectroscopy, which is a measure of cumulative PrEP adherence over several weeks (24). We defined objective PrEP persistence as any TFV-DP or “PrEP” concentration levels detected in the collected DBS samples at the follow-up study visit (3 and 6 months) from those who initiated PrEP at baseline and those with DBS samples that were collected and analyzed. The DBS samples were analyzed for a non-random sample of TFV-DP measures of the first 900 participants of the full cohort (*n* = 1,195) among those who reported using PrEP in the last 30 days of the study visit.

As recommended by the pharmacokinetic study by Stranix-Chibanda et al. (24), we used separate thresholds for adherence using TFV-DP in DBS in pregnant vs. postpartum women. High adherence or daily intake oral PrEP (∼7 doses/week) was defined by DBS with a TFV-DP value of ≥600 fmol/punch for pregnant women and ≥1,000 fmol/punch for postpartum women; moderate adherence (2–6 doses/week) was defined as DBS with a TFV-DP value of 200–599 fmol/punch for pregnant women and 400–999 fmol/punch for postpartum women; and low adherence (<2 doses/week) was defined as quantifiable with a TFV-DP value of <200 fmol/punch for pregnant women and <400 fmol/punch for postpartum women. We then classified them as high, moderate, low, and below the quantifiable TFV-DP concentrations. Due to the low number of women with high TFV-DP (∼7 doses/week), this outcome compared women with quantifiable TFV-DP concentrations with those with unquantifiable TFV-DP concentrations. We also included those who did not report taking PrEP in the last 30 days as part of the denominator and classified them as non-adherent. However, those who reported recent adherence, but did not have DBS analyzed, were marked as missing from the analysis because their adherence levels were unknown.

### Exposure: HIV risk score and risk perception

The two primary exposures of this study were baseline HIV risk score and risk perception for HIV. We created a composite baseline risk score based on the number of behavioral HIV risk factors reported (range 0–5), which was adapted from another study examining HIV risk among AGYW (17). The HIV risk score is a sum of five factors that are scored at 1 point each: condomless sex, having more than one sexual partner, having a primary partner living with HIV or with unknown serostatus, laboratory-confirmed STI diagnosis at baseline, and hazardous alcohol use [Alcohol Use Disorders Identification Test for Consumption (AUDIT-C) score ≥ 3] in the year prior to pregnancy. We used this risk score as a continuous variable and created a two-category HIV risk variable (≤1 and ≥2) to examine the differences between lower and higher risk scores. We defined risk perception as answering either “no chance,” “low chance,” or “high chance” to the question “How would you describe your chances of getting HIV in the next year?” at baseline.

### Covariates

Relevant demographic measures included the highest level of education, socioeconomic status, gravidity, and relationship status, which were collected by a study interviewer at each study visit using a survey on REDCap. Clinical characteristic measures were gestational age in weeks at the first ANC visit. Baseline STI diagnosis was determined based on results from a self-collected vaginal swab tested for *Chlamydia trachomatis* (CT), *Neisseria gonorrhoeae* (NG), and *Trichomonas vaginalis* (TV) (Cepheid Inc., Sunnyvale, CA, USA).

At baseline, the participants were asked with regard to the number of sexual activity, condom use during the last sex, number of sexual partners in the past 12 months, HIV status of the partner in the past 12 months, intimate partner violence (IPV) in the past 12 months (WHO IPV scale) (25), and alcohol use in the past 12 months and before finding out about their pregnancy using the AUDIT-C (26). Alcohol use was defined as reporting any alcohol use or by a cutoff of AUDIT-C score of ≥3, which was used in our previous study to identify hazardous alcohol use among pregnant women in South Africa (20). We also reported pregnancy status at 1, 3, and 6 months and HIV risk perception and number of sex acts at 3 and 6 months.

### Statistical analysis

We restricted the analytical sample of this study to participants aged 16–24 years at baseline (*n* = 486). First, the baseline characteristics were described overall and stratified by age categories of adolescent girls (aged 16–18 years) and young women (aged 19–24 years). We reported the median [interquartile range (IQR)] for the continuous variables and frequency/percentage for the categorical variables. We then compared the baseline characteristics by age using Student’s *t*-test, chi-squared test, or Fisher's exact test. We used Fisher's exact test, which uses the data directly, when we had cell counts of <10 as a conservative measure instead of the chi-squared test, which only relies on an approximation (27).

We evaluated the PrEP cascade by estimating the proportion of AGYW who were eligible for, initiated, and continued PrEP use (1, 3, and 6 months). We censored those who experienced pregnancy loss and infant loss or those whose HIV status changed to positive during the study follow-up; these participants were removed from the denominators during follow-up. The cascade was shown as overall, by their HIV risk scores, and by sub-age categories. Finally, we ran the crude and adjusted logistic regression models to estimate odds ratios for the association between behavioral HIV risk factors and PrEP outcomes using separate models. We reported the associated 95% CI for each model. In the adjusted analyses, we controlled for maternal age and gestational age at baseline and whether the baseline data were collected before or during/after the national COVID-19 lockdowns in South Africa (before/after 28 March 2020) (28). All analyses were performed using SAS version 9.4 (SAS Institute).

## Results

### Patient characteristics

Out of the 1,195 women enrolled in the PrEP-PP study, 486 were AGYW. Specifically, 16% (*n* = 77) were “adolescents” aged 16–18 years, and 84% (*n* = 409) were “young women” aged 19–24 years (Table 1). In total, 67% (*n* = 327) were pregnant with their first child, and 70% (*n* = 340) were neither married nor cohabitating with their partner. Compared with young women, more adolescent girls were neither married nor cohabiting with their partners (78% vs. 68%, *p *= 0.01).

**Table 1:** Baseline characteristics of pregnant adolescent girls and young women (aged 16–24 years at enrollment) from the PrEP-PP study in Cape Town, South Africa (*N* = 486).

	Overall	Age (16–18 years)	Age (19–24 years)	*p*-value
	*n* (%)	*n* (%)	*n* (%)	
Total	486 (100)	77 (16)	409 (84)	
Demographics				
Highest level of education				
<Grade 12	209 (43)	54 (70)	155 (38)	**<0**.**001**
≥Grade 12	277 (57)	23 (30)	254 (62)	
Socioeconomic status				
Low SES	160 (33)	33 (43)	127 (31)	**0**.**04**
Moderate to high SES	326 (67)	44 (57)	282 (69)	
Gravidity				
Primigravida	327 (67)	70 (91)	257 (63)	**<0**.**001**
Multigravida	159 (33)	7 (9)	152 (37)	
Relationship status				
Neither married nor cohabiting	340 (70)	60 (78)	280 (68)	**0**.**01**
Married or cohabitating	90 (19)	5 (6)	85 (21)	
Not in a relationship	56 (12)	12 (16)	44 (11)	
Clinical characteristics				
Gestational age at first ANC visit, weeks (median, IQR)	24 (17–34)	28 (20–34)	23 (16–34)	**0**.**04**
Gestational age at first ANC visit, weeks				
≤14	95 (20)	9 (12)	86 (21)	0.06
>14	391 (80)	68 (88)	323 (79)	
Any STI diagnosed (CT, NG, and/or TV)				
No STI	283 (59)	35 (46)	248 (61)	**0**.**01**
STI diagnosed	200 (41)	41 (54)	159 (39)	
Type of STI diagnosed (CT, NG, and/or TV)				
CT only	120 (60)	22 (54)	98 (62)	NE
NG only	13 (7)	0 (0)	13 (8)	
TV only	16 (8)	4 (10)	12 (8)	
CT and NG	29 (15)	11 (27)	18 (11)	
CT and TV	16 (8)	2 (5)	14 (9)	
TV and NG	5 (3)	1 (2)	4 (3)	
CT, NG, and TV	1 (1)	1 (2)	0 (0)	
Behavioral risk factors				
Sexually active in pregnancy (at baseline)				
Not sexually active	17 (4)	4 (5)	13 (3)	**0**.**04**
Yes, 1–4 times per month	303 (62)	56 (73)	247 (60)	
Yes, 5+ times per month	166 (34)	17 (22)	149 (36)	
Condom use during last sex (at baseline)^a^				
Condomless sex	291 (62)	42 (58)	249 (63)	0.39
Condom used	178 (38)	31 (42)	147 (37)	
Number of sexual partners in the past 12 months				
One sexual partner	392 (81)	63 (82)	329 (80)	0.78
>1 sexual partners	94 (19)	14 (18)	80 (20)	
Partner’s HIV status in the past 12 months (at baseline)^a^				
HIV-negative	319 (66)	47 (61)	272 (67)	0.40
HIV-positive	4 (1)	0 (0)	4 (1)	
Do not know	163 (34)	30 (39)	133 (33)	
IPV in the past 12 months^b^				
No IPV	430 (88)	68 (88)	362 (89)	0.96
Reported IPV	56 (12)	9 (12)	47 (11)	
Alcohol use in the past 12 months before pregnancy				
No alcohol use	217 (45)	40 (52)	177 (43)	0.16
Any alcohol use	269 (55)	37 (48)	232 (57)	
Hazardous Alcohol use (AUDIT-C ≥ 3)	167 (34)	23 (30)	144 (35)	0.37
Baseline HIV risk^c^ (dichotomized)				
No/low HIV risk (score ≤ 1)	179 (37)	29 (38)	150 (37)	0.87
Moderate/high HIV risk (score > 1)	307 (63)	48 (62)	259 (63)	
Baseline HIV risk score^c^				
0	44 (9)	9 (12)	35 (9)	0.52
1	135 (28)	20 (26)	115 (28)	
2	172 (35)	23 (30)	149 (36)	
3	105 (22)	18 (23)	87 (21)	
4	25 (5)	5 (6)	20 (5)	
5	5 (1)	2 (3)	3 (1)	

Bold values represent *p*-values <0.05.

NE, not estimated; Data are *n* (%) or median (IQR).

^a^ In women who reported sexual partners.

^b^ The participants were considered to have experienced any IPV if they endorsed at least one of four items asking about their recent physical, emotional, or sexual violence from a sexual partner.

^c^ HIV risk score is the sum of points for reporting each of the following risk factors at baseline: condomless sex, >1 sexual partner, partner living with HIV or unknown partner HIV status, laboratory-confirmed STI diagnosis at baseline, and hazardous alcohol use.

### Clinical characteristics

The overall median gestational age during the first ANC visit was 24 weeks (IQR = 17–34) (Table 1). The median gestation age at baseline for adolescent girls was later [28 (20–34) weeks] when compared with that for young women [23 (16–34) weeks, *p *= 0.04]. Thus, more adolescents attended ANC visits for the first time at over the recommended 14 weeks of gestation compared with young women (88% vs. 79%, *p *= 0.06). Moreover, almost all the adolescent girls were primigravida compared with the proportion of young women (91% vs. 63%, *p *< 0.01). Over half (54%, *n* = 41) of the adolescent girls were diagnosed with STI at baseline compared with the 39% (*n* = 159) of the young women. Adolescent girls also presented with multiple sexually transmitted co-infections compared with young women (19% vs. 9% with multiple STIs, respectively).

### Behavioral risk factors

Of the 96% (*n* = 469) of AGYW who were sexually active at baseline, the majority (62%, *n* = 291) practiced condomless sex at baseline, and most (81%, *n* = 392) reported having only one sexual partner (Table 1). Overall, 66% (*n* = 319) reported having partners who were not living with HIV, 34% (*n* = 163) reported that they did not know their partner's HIV status, and 1% (*n* = 4) reported that their partner is living with HIV at baseline. Approximately 12% of AGYW reported experiencing intimate partner violence in the past 12 months, and over half (55%, *n* = 269) reported alcohol use in the last 12 months before pregnancy. Prior to their pregnancy, 34% (*n* = 167) reported hazardous alcohol use (AUDIT-C ≥ 3).

Most behavioral risk factors, such as condom use during sex (42% vs. 37%, *p *= 0.39), multiple sexual partners (18% vs. 20%, *p *= 0.78), knowledge regarding their partner's HIV status in the past 12 months (61% vs. 67%, *p* = 0.40), and composite HIV risk score (62% vs. 63% scoring 2+, *p *= 0.83), were similar among AGYW.

### PrEP cascade in pregnant and postpartum AGYW

**Figure 1:**
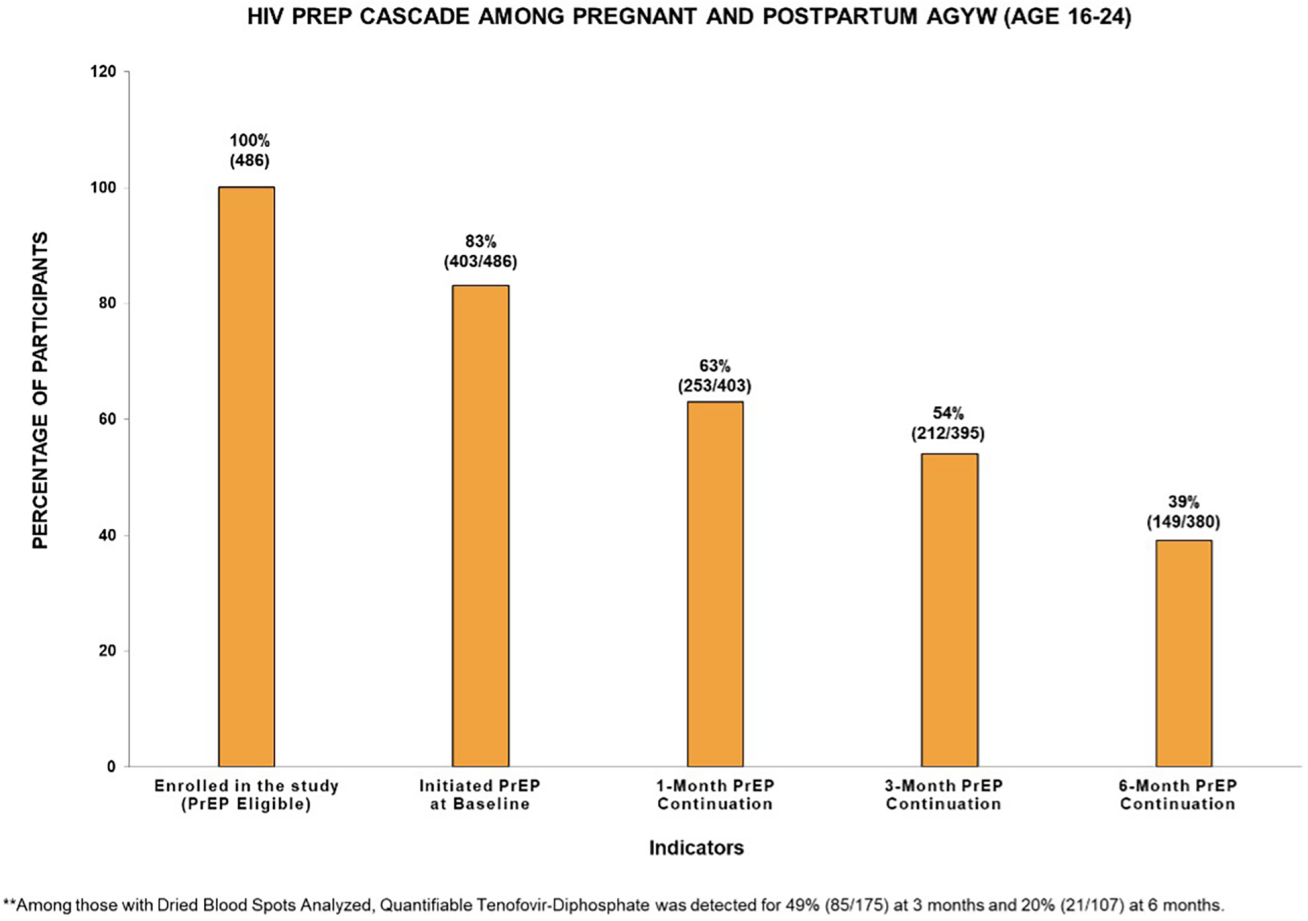
HIV PrEP cascade among pregnant and postpartum adolescent girls and young women (aged 16–24 years) in the PrEP-PP study (*n* = 486).

Figure 1 displays the HIV PrEP cascade indicators among pregnant and postpartum AGYW at baseline and at 1-, 3-, and 6-month follow-up visits. Of the 83% (403/486) who initiated PrEP, the percentage of continuation during follow-up was 63% (253/403) at 1 month, 54% (212/395) at 3 months, and 39% (149/380) at 6 months. These AGYW had similar PrEP uptake and continuation prevalence across the cascade (Supplementary Figure S1). Approximately 27% (110/403) consistently attended all visits through 6 months after initiating PrEP, and 6% (25/408) missed either the 1- or 3-month visit but restarted on PrEP at 6 months (Table 2). Among those who restarted on PrEP, most were postpartum women at 6 months (80%, *n* = 20). Of those with DBS samples collected and analyzed, a quantifiable TFV-DP concentration was detected among 49% (85/175) at 3 months and 20% (21/107) at 6 months. Disaggregating adherence data further, 6% (*n* = 10) were found to have high adherence (∼7 doses/week), 20% (*n* = 35) had medium adherence (∼2–5 doses/week), 23% (*n* = 40) had low adherence (<2 doses/week), and 51% (*n* = 90) had unquantifiable TFV-DP concentration levels or did not report using PrEP in the last 30 days at the 3-month visit. Meanwhile, 1% (*n* = 1) were found to have high adherence (∼7 doses/week), 5% (*n* = 5) had medium adherence (∼2–5 doses/week), 14% (*n* = 15) had low adherence (<2 doses/week), and 80% (*n* = 80) had unquantifiable TFV-DP concentration levels or did not report using PrEP in the last 30 days of the 6-month visit.

**Table 2:** The PrEP cascade indicators among pregnant and postpartum adolescent girls and young women (aged 16–24 years) from the PrEP-PP study in Cape Town, South Africa (*N* = 486).

	Overall	Age (16–18 years)	Age (19–24 years)	*p*-value
	*n* (%)	*n* (%)	*n* (%)	
Baseline				
Total at baseline	486 (100)	77 (16)	409 (84)	
HIV risk perception at baseline				
No chance	283 (58)	48 (62)	235 (57)	**0**.**04**
Low chance	161 (33)	18 (23)	143 (35)	
High chance	42 (9)	11 (14)	31 (8)	
Sexually active at baseline				
Not sexually active	17 (4)	4 (5)	13 (3)	**0**.**04**
Yes, 1–4 times per month	303 (62)	56 (73)	247 (60)	
Yes, 5+ times per month	166 (34)	17 (22)	149 (36)	
PrEP initiation at baseline				
Did not initiate PrEP	83 (17)	13 (17)	70 (17)	0.96
Initiated PrEP at baseline	403 (83)	64 (83)	339 (83)	
1-month follow-up				
Total at the 1-month follow-up				
Attended and continued PrEP	253 (63)	38 (59)	215 (63)	NE
Attended and discontinued PrEP	23 (6)	3 (5)	20 (6)	
Missed visit and discontinued PrEP	119 (24)	22 (29)	97 (24)	
Initiated PrEP at 1-month visit	1 (0.2)	0 (0)	1 (0.2)	
Never on PrEP	81 (17)	13 (17)	68 (17)	
Censored	9 (2)	1 (2)	8 (2)	
Pregnancy status at 1 month				
Pregnant	314 (78)	47 (75)	267 (79)	0.49
Postpartum	89 (22)	16 (25)	73 (21)	
3-month follow-up				
Total at the 3-month follow-up				
Attended and continued PrEP	176 (40)	28 (37)	148 (41)	NE
Attended and discontinued PrEP	21 (5)	0 (0)	21 (6)	
Missed visit and discontinued PrEP	21 (4)	21 (28)	126 (31)	
Restarted PrEP	37 (8)	11 (15)	26 (7)	
Initiated PrEP at 3-month visit	16 (3)	0 (0)	16 (4)	
Never on PrEP	62 (13)	13 (17)	49 (12)	
Censored	18 (4)	3 (4)	15 (4)	
Pregnancy status at 3 months				
Pregnant	238 (51)	25 (34)	213 (54)	**<0**.**01**
Postpartum	226 (49)	48 (66)	178 (46)	
HIV risk perception at 3 months				
No chance	153 (55)	22 (55)	131 (55)	0.41
Low chance	103 (37)	13 (33)	90 (38)	
High chance	21 (8)	5 (13)	16 (7)	
Sexually active at 3 months				
Not sexually active	89 (32)	22 (55)	67 (28)	**<0**.**01**
Yes, 1–4 times per month	143 (52)	14 (35)	129 (54)	
Yes, 5+ times per month	45 (16)	4 (10)	41 (17)	
Sexually active while pregnant	135 (84)	12 (75)	123 (85)	0.28
Sexually active while postpartum	53 (45)	6 (25)	47 (51)	**0**.**04**
PrEP persistence at 3 months (30-day self-report)				
High adherence (∼7 days)	134 (57)	20 (51)	114 (51)	0.81
Medium adherence (2–6 days)	53 (23)	9 (23)	44 (20)	
Low adherence (<2 doses/week)	4 (2)	1 (3)	3 (2)	
Did not adhere to PrEP	44 (19)	9 (23)	35 (18)	
PrEP persistence at 3 months (TFV-DP)				
High adherence (∼7 days)	10 (6)	1 (3)	9 (6)	0.52
Medium adherence (2–6 days)	35 (20)	9 (29)	26 (18)	
Low adherence (>BLQ)	40 (23)	7 (23)	33 (23)	
BLQ or reported not using PrEP	90 (51)	14 (45)	76 (53)	
PrEP persistence at 3 months (TFV-DP)				
Any TFV-DP concentration levels detected	85 (49)	17 (55)	68 (47)	0.44
BLQ or reported not using PrEP	90 (51)	14 (45)	76 (53)	
6-month follow-up				
Total at the 6-month follow-up				
Attended and continued PrEP	132 (30)	26 (37)	106 (29)	NE
Attended and discontinued PrEP	35 (8)	2 (3)	33 (9)	
Missed visit and discontinued PrEP	181 (39)	28 (38)	153 (40)	
Restarted PrEP	25 (6)	3 (4)	22 (6)	
Initiated PrEP at 6-month visit	4 (0.9)	0 (0)	4 (1)	
Never on PrEP	51 (11)	11 (15)	40 (10)	
Censored	31 (7)	3 (4)	28 (8)	
Pregnancy status at 6 months				
Pregnant	83 (19)	4 (6)	79 (21)	**0**.**001**
Postpartum	357 (81)	66 (94)	291 (79)	
HIV risk perception at 6 months				
No chance	125 (57)	17 (52)	108 (58)	0.74
Low chance	86 (39)	15 (45)	71 (38)	
High chance	7 (3)	1 (3)	6 (3)	
Sexually active at 6 months				
Not sexually active	62 (28)	9 (27)	53 (29)	0.62
Yes, 1–4 times per month	118 (54)	20 (61)	98 (53)	
Yes, 5+ times per month	38 (17)	4 (12)	34 (18)	
Sexually active while pregnant	51 (94)	2 (100)	49 (94)	NE
Sexually active while postpartum	105 (64)	22 (71)	83 (62)	0.41
PrEP persistence at 6 months (30-day self-report)				
High adherence (∼7 days)	87 (48)	17 (55)	70 (47)	0.53
Medium adherence (2–6 days)	44 (24)	7 (23)	37 (25)	
Low adherence (<2 doses/week)	3 (2)	1 (3)	2 (1)	
Did not adhere to PrEP	47 (26)	6 (19)	41 (27)	
PrEP persistence at 6 months (TFV-DP)				
High adherence (∼7 days)	1 (1)	0 (0)	1 (1)	NE
Medium adherence (2–6 days)	5 (5)	2 (11)	3 (3)	
Low adherence (>BLQ)	15 (14)	2 (11)	13 (15)	
BLQ or reported not using PrEP	86 (80)	15 (79)	71 (81)	
PrEP persistence at 6 months (TFV-DP)				
Any TFV-DP concentration levels detected	21 (20)	4 (21)	17 (19)	1.00
BLQ or reported not using PrEP	86 (80)	15 (79)	71 (81)	
Continued PrEP through the 6-month visit^a^	110 (27)	22 (34)	88 (26)	0.17

Bold values represent *p*-values <0.05.

BLQ, below the limit of quantification; NE, not estimated.

^a^ Continued consistently through the 6-month visit are those who attended all study visits (1-, 3-, and 6-month follow-up) among those who initiated PrEP at baseline.

Most pregnant AGYW (96%) were sexually active at baseline. Of those who continued participating in the study, 68% were sexually active at the 3-month follow-up, and 71% were sexually active at the 6-month follow-up (Table 2). At the 3-month visit, 51% (*n* = 238) were pregnant, and 49% (*n* = 226) were postpartum women. A higher proportion of adolescent girls reported being sexually abstinent during the postpartum period compared with young women (75% vs. 52%, *p *= 0.04). However, at 6 months, the frequency of sexual activity during the postpartum period was similar among AGYW (31% vs. 32%, *p *= 0.89). At 3 months, most AGYW had sex while pregnant (84%, *n* = 135), whereas fewer AGYW had sex during the postpartum period (45%, *n* = 53). However, the young women reported being more sexually active during the postpartum period than the adolescent girls (51% vs. 25*%, p *= 0.04)*.* At 3 months, the adolescent girls who reported no perceived HIV risk also reported sexual abstinence (55%, *n* = 12); meanwhile, approximately 55% (*n* = 131) of the young girls reported no perceived HIV risk, and only 28% (*n* = 37) reported sexual abstinence.

Supplementary Figure S2 displays the PrEP cascade indicators among pregnant and postpartum AGYW at baseline and at 1-, 3-, and 6-month follow-up visits stratified by their baseline HIV risk scores (≤1 and ≥2). Although the proportions of AGYW who initiated PrEP at baseline were similar (82% vs. 83%, *p *= 0.72), the percentage of continuation at 1 month (56% vs. 66%, *p *= 0.05), 3 months (45% vs. 59%, *p *= 0.01), and 6 months (34% vs. 42%, *p *= 0.09) was higher among those with greater HIV baseline risk scores compared with the percentage among those with lower risk scores. However, the proportion of those with any TFV-DP concentration levels detected in the blood was similar at 3 months (48% vs. 49%, *p *= 0.84) and 6 months (14% vs. 22%, *p* = 0.43).

Table 3 summarizes the associations between HIV risk score, risk perception, and outcomes from the PrEP cascade. Frequencies for these associations can be found in Supplementary Table S1. AGYW with a higher risk score (≥2) showed higher odds of PrEP continuation at 3 months [adjusted odds ratio (aOR): 1.60 (95% CI: 1.05–2.43)] and consistent PrEP continuation through 6 months [aOR: 1.85 (95% CI: 1.12–3.03)], after adjusting for maternal age and gestational age at baseline and whether the baseline data was collected before/after 28 March 2020 (national COVID-19 early pandemic lockdowns). Compared with AGYW who perceived no HIV risk at baseline, those with high HIV risk perception showed higher adjusted odds of PrEP continuation at 3 months [aOR: 1.90 (95% CI: 0.88–4.13)] and consistent PrEP continuation through 6 months [aOR: 1.89 (95% CI: 0.89–4.00)], although the confidence intervals crossed the null.

**Table 3:** HIV risk factors, risk perception and outcomes from the PrEP Cascade among pregnant and postpartum Adolescent Girls and Young Women (aged 16 to 24 years) from the PrEP-PP study in Cape Town, South Africa.

	PrEP status, Odds Ratio (95% CI)						
	Initiation (Baseline)	Continuation (1-month)	Continuation (3-month)	Continuation (6-month)	Continued through 6 months	Any TFV-DP at 3-month	Any TFV-DP at 6-month
Crude							
Baseline HIV Risk Score (continuous)	1.03 (0.82, 1.28)	1.26 (1.04, 1.53)	1.21 (1.01, 1.46)	1.11 (0.91, 1.34)	1.31 (1.06, 1.61)	1.24 (0.94, 1.62)	1.58 (0.98, 2.57)
Baseline HIV Risk Score No/Low HIV risk (score .1) Moderate/High HIV risk (score .2)	Reference 1.09 (0.67, 1.78)	1.52 (1.01,2.31)	1.72 (1.14, 2.59)	1.44 (0.93, 2.22)	2.00 (1.23, 3.26)	1.07 (0.57, 1.99)	1.71 (0.57, 5.14)
HIV Risk Perception at baseline							
No Chance	Reference						
Low Chance	0.85 (0.51, 1.41)	1.32 (0.85, 2.07)	1.29 (0.83, 1.98)	1.16 (0.74, 1.83)	1.14 (0.71, 1.85)	1.30 (0.68, 2.49)	NE
High Chance	0.97 (0.41, 2.32)	1.46 (0.68, 3.12)	2.18 (1.02, 4.66)	1.71 (0.83, 3.53)	2.26 (1.09, 4.7)	0.82 (0.30, 2.22)	NE
Adjusted*							
Baseline HIV Risk Score (continuous)	1.05 (0.84, 1.31)	1.25 (1.03, 1.51)	1.19 (0.98, 1.43)	1.09 (0.89, 1.32)	1.29 (1.04, 1.59)	1.21 (0.91, 1.59)	1.62 (0.98, 2.66)
Baseline HIV Risk Score***							
No/Low HIV risk (score .1)	Reference						
Moderate/High HIV risk (score .2)	1.18 (0.72, 1.93)	1.47 (0.96, 2.23)	1.60 (1.05, 2.43)	1.32 (0.85, 2.06)	1.85 (1.12, 3.03)	1.03 (0.54 1.93)	1.73 (0.56, 5.40)
HIV Risk Perception at baseline							
No Chance	Reference						
Low Chance	0.89 (0.53, 1.50)	1.31 (0.84, 2.06)	1.29 (0.83, 2.02)	1.15 (0.72, 1.83)	1.11 (0.68, 1.82)	1.41 (0.73, 2.75)	NE
High Chance	1.24 (0.51, 3.00)	1.35 (0.63, 2.91)	1.90 (0.88, 4.13)	1.42 (0.67, 3.00)	1.89 (0.89, 4.00)	0.93 (0.34, 2.57)	NE

Abbreviations: PrEP, pre-exposure prophylaxis; aOR, adjusted Odds Ratio; TFV-DP = tenofovir disoproxil fumarate/emtricitabine; CT = Chlamydia trachomatis, NG = Neisseria gonorrhoeae, PrEP = pre-exposure prophylaxis, STI = sexually transmitted infection, TV = Trichomonas vaginalis, IPV = Intimate Partner Violence

Bold: statistically significant measures that do not cross the null (1.00) N(%) for this table can be found in Supplemental Table 1 NE = Not estimated due to insufficient sample size for a logistic regression.

*adjusted for maternal age, gestational age at baseline, and whether baseline data was collected before or during/after the national COVID-19 pandemic lockdowns in South Africa (defined as before/after March 28, 2020)

***HIV risk score is a sum of points for reporting each of the following risk factors at baseline: condomless sex, reporting >1 sexual partner, reporting of a partner living with HIV or unknown partner HIV status, laboratory-confirmed STI diagnosis at baseline, reporting hazardous alcohol use

Outcome definitions: a) initiation (baseline) are those who initiated PrEP among those PrEP eligible at baseline visit (n = 486); b) continuation at 1 are those who attended and requested a PrEP prescription among those who initiated PrEP at baseline (n = 403); c) continuation at 3, 6 months are those who attended and requested a PrEP prescription among those who initiated PrEP at baseline and removed those who were censored for pregnancy/infant loss or HIV seroconversion (n = 395 for 3-month and n = 380 for 6-month); c) continued consistently to 6 months visit are those who attended all study visits (1-,3-, and 6-month follow-up) among those who initiated PrEP at baseline (n = 403); d) persisted on PrEP is any TFV-DP detected among those who reported PrEP use in the last 30 days and had dried blood spots analyzed. Those who reported not using PrEP in the last 30 days were marked as “did not adhere” (n = 175 at 3 months and n = 107 at 6 months).

Compared with those with no STI at baseline, pregnant AGYW diagnosed with STI at baseline had 1.5 times the adjusted odds of PrEP continuation at 1 month [STI: aOR: 1.46 (95% CI: 0.96–2.24)], and a similar association was observed for those with consistent PrEP continuation through 6 months [STI diagnosed: aOR: 1.28 (95% CI: 0.81–2.03)] (Supplementary Table S4). Frequencies for these associations can be found in Supplementary Table S2. Compared with those with no alcohol use at baseline, AGYW who reported alcohol use had slightly higher odds of PrEP continuation at 3 months [alcohol use: aOR: 1.65 (95% CI: 1.10–2.49)] and 6 months [alcohol use: aOR: 1.41 (95% CI: 0.92–2.17)] and consistent PrEP continuation through 6 months [alcohol use: aOR: 1.51 (95% CI: 0.96–2.40)]. AGYW with a partner living with HIV or with unknown serostatus also had slightly higher adjusted odds of consistently continuing PrEP through 6 months compared with those with a partner not living with HIV [partner living with HIV or with unknown serostatus: aOR: 1.40 (95% CI: 0.88–2.21)].

## Discussion

In this cohort study of 486 pregnant and postpartum AGYW, we observed high overall PrEP initiation (>80%). However, at 6 months, only just over one-third of those who initiated PrEP continued. Meanwhile, among those who discontinued, 6% of AGYW restarted on PrEP use at 6 months. PrEP continuation was higher among those with greater baseline HIV risk scores and higher perceived HIV risk. Moreover, we identified important age-specific clinical characteristics between pregnant and postpartum AGYW in our study. This study also contributed to the paucity of literature on the PrEP cascades among the pregnant/postpartum AGYW from health facilities in South Africa.

### Clinical characteristics of AGYW at baseline

Most adolescent girls (aged <19 years) attended their first ANC visit much later at 28 weeks of gestation, which is in the third trimester of the pregnancy. This differs from our previous study among the overall PrEP-PP samples with older women, where the median gestation at ANC initiation was 21 weeks (second trimester). Although this timing is still later than what is recommended in the WHO guideline for women to initiate ANC, which is approximately 12 weeks (first trimester) (29), and in the national South African guidelines, which is 14 weeks, it supports the findings of previous studies that reported that AGYW access ANC much later than older women in sub-Saharan Africa (30). The early timing of initiating ANC is particularly important in HIV prevention methods as this could impact access to early PrEP initiation for those at risk, HIV diagnosis, and early HIV treatment.

Moreover, over half (54%) of the adolescent girls in our sample were diagnosed with STI at the baseline visit, often presented with multiple sexually transmitted co-infections (19%). STI case management is typically performed at a primary care setting, and for AGYW, the 2022 guidelines by the Southern African HIV Clinicians Society recommended that STI screening should be conducted at least once a year based on the assessment of risk factors (e.g., multiple sex partners, engagement in transactional sex, sex under the influence of drugs, or STI diagnosis in the past year) (31). Given the late ANC initiation and high STI burden among adolescent girls in our study, HIV prevention methods should promote early ANC visits and strengthen interventions to actively test, manage, and treat STIs beyond a primary care setting (32, 33).

### PrEP initiation and continuation

The prevalence of PrEP initiation in our study was comparable with that of other studies on AGYW in sub-Saharan Africa (34). Unlike other studies that reported lower PrEP uptake for AGYW (34, 35), PrEP uptake in our study (83%) was similar to the finding of the overall PrEP-PP study with older women (84%) (21), and we also did not observe any differences between the age groups of AGYW. Although the percentage of PrEP continuation in our sample was low (63% at 1 month, 54% at 3 months, and 39% at 6 months), it was higher compared with that of similar studies on AGYW (32% at 1 month and 6% at 3 months) in Kenya (34). Both studies had oral PrEP-focused projects among AGYW; however, our study was comprised of only pregnant AGYW who were regularly coming in for their prenatal care, because non-pregnant AGYW had no reason to return to clinics solely for PrEP.

### HIV risk score, risk perception, and PrEP continuation

In our analysis, the continuation of PrEP differed by baseline HIV risk score and by self-perceived HIV risk. The risk scores had previously been used to identify those at high risk of acquiring HIV (36, 37). We used a modified risk score to fit the data available in our study and to reflect the relevant clinical and behavioral factors (e.g., condomless sex, more than one sexual partner, primary partner living with HIV or unknown serostatus, STI diagnosis at baseline, and hazardous alcohol use). Despite the low overall percentage of continuation, we found that those with greater HIV risk had higher odds of consistent PrEP continuation through 6 months. We also found that having a high-risk perception at baseline was correlated with higher odds of consistent PrEP continuation PrEP. Although the risk scores are objectively calculated on a series of sexual behaviors and risk perception is seemingly subjective, studies have found the concepts overlapping (38). Hensen et al. reported that AGYW made decisions on PrEP use based on their HIV risk perception, including condom use, number of sexual partners, and being married/cohabitating with a partner (38), all of which were used to develop our risk score. Prior studies also reported that AGYW who initiated PrEP were motivated by a high perceived HIV risk (34). The risk score at baseline may be used to objectively identify those that would benefit the most from HIV prevention methods, such as PrEP.

### PrEP persistence (tenofovir levels in DBS)

PrEP persistence, measured using DBS to detect the presence of TFV-DP, was only examined among a systematic subset of women who reported PrEP use in the last 30 days. The proportion of those with quantifiable TFV-DP concentration levels was low (49% at 3 months and 20% at 6 months). Due to this finding, a limitation of our study was that this may be under- or overreporting the true proportion of women taking oral PrEP. However, similar to prior studies, we measured the quantifiable vs. unquantifiable TFV-DP concentration levels in our analysis since the number of AGYW with high TFV-DP concentration levels, consistent with ∼7 doses per week, was small (6% at 3 months and 1% at 6 months) (17). We remain concerned that the tenofovir concentration levels in our sample were low and inadequate for ample HIV protection even among those reporting recent PrEP use. A strength of our analysis was using a biomarker (i.e., TFV-DP levels in the blood) to measure persistence over self-reported adherence, which correlated poorly with each other in our previous study (22).

### Adherence challenges among pregnant/postpartum AGYW

Qualitative assessments among AGYW have described that PrEP persistence is difficult with dwindling motivations for taking a preventative pill while being healthy, citing the daily pill burden (size and frequency) (21, 39, 40) and stigma with taking the pill (19, 39, 40). Meanwhile, others indicated the benefits of PrEP, citing that they feel safer while on the pill especially with changing risks (39). In our earlier analysis, we also reported that side effects such as nausea and vomiting may overlap with pregnancy symptoms, which guided PrEP counseling in the clinics (21). Prior literature has indicated that PrEP adherence may be less among pregnant women due to pregnancy itself and waning could occur during the postpartum periods (22), which was also observed among the AGYW in our study.

We also recognized that evaluating prevention-effective adherence, which aligns changing HIV risk with PrEP adherence levels, is important in PrEP studies (17, 41, 42). Studies have reported that women, including AGYW, may already be starting and stopping PrEP with changing risks (16). Approximately 6% of the AGYW in our study stopped and restarted PrEP at 6 months, and most (80%) of them were postpartum women. However, we were unable to examine changing sexual risks because those who discontinued PrEP use also missed attending follow-up study visits. Therefore, we were unable to obtain the changing HIV risk information among those who discontinued participating in the study. Given that this is a sample of pregnant and postpartum women across different gestational weeks, future studies could examine whether there are patterns in sexual activity (vs. abstinence) by gestational weeks and examine prevention-effective adherence to PrEP by gestational timing. Future interventions could include concepts such as community PrEP delivery in pharmacies or community pick-up points for postpartum women, biofeedback using accurate reflections of PrEP use to align with changing sexual risks (using urine or other tenofovir testing), and peer support models for PrEP. Moreover, implementing the long-acting injectable PrEP (cabotegravir) may improve HIV protection for pregnant/postpartum AGYW. Studies have reported that pregnant and postpartum women who have used oral PrEP showed a theoretical preference for long-acting injectable PrEP (43), and this finding may help address notable barriers to sufficient HIV protection due to adherence challenges (44). Future trials on long-acting injectable PrEP should include pregnant/postpartum populations and AGYW so that cabotegravir can be widely implemented among those at high risk of acquiring HIV.

### Limitations

Our study has a few limitations. First, we analyzed a non-random sample of TFV-DP measures among the first 900 participants (when the study budget was available) of the cohort who reported using PrEP in the last 30 days. Thus, for those who reported using PrEP, but did not have their DBS analyzed later in the study, we marked their adherence levels as unknown/missing. Second, the low adherence to PrEP among AGYW could be due to the changing HIV risks. Since many of the AGYW are visiting the clinic closer to their delivery date, they may not be sexually active during their late pregnancy or early postpartum period. We were unable to examine the changing HIV risks due to the collinearity between those who discontinued PrEP use and missed attending the study visits. Third, the PrEP-PP study data were collected from one urban ANC clinic in Cape Town, South Africa, which may not be generalizable to other geographical regions or populations. Fourth, given that the surveys were administered by study staff at a health clinic on sensitive information such as sexual behaviors, intimate partner violence, and alcohol use among AGYW, errors might be reported due to social desirability bias. However, we used the biomarkers when feasible, such as STI diagnosis at baseline and DBS, to measure the TFV-DP concentration levels.

## Conclusion

Using the PrEP cascade for pregnant and postpartum AGYW accessing ANC in South Africa, we found a high percentage of PrEP initiation but retained just over one-third of the sample by 6 months. High HIV risk score and high-risk perception were both associated with increased odds of continuing PrEP through 6 months. However, even among AGYW reporting consistent PrEP use, only 20%–49% had detectable TFV-DP concentration levels, which means that PrEP coverage remained inadequate for ample HIV protection. Moreover, pregnant AGYW initiated ANC visits much later, with a high burden of untreated STIs. These findings suggest the existence of key barriers in HIV prevention methods for AGYW during pregnancy and postpartum periods. Based on our findings, we recommend integrating HIV and PrEP counseling, including longer-acting treatments when they become available, into ante- and postpartum care and community delivery to de-medicalize and simplify PrEP delivery among pregnant and breastfeeding women.

## Data Availability

The data sets presented in this article are not readily available. Requests to access the data sets should be directed to DJosephDavey@mednet.ucla.edu.
