# Supplementary material for: Evaluating the use of oral pre-exposure prophylaxis among pregnant and postpartum adolescent girls and young women in Cape Town, South Africa
Source: Front Reprod Health. 2023 Sep 19;5:1224474. doi: 10.3389/frph.2023.1224474 (PMC10546059; doi:10.3389/frph.2023.1224474)
Supplement: Supplementary file 1 [file Datasheet1.docx]

| **Supplemental Table 1. HIV risk factors and risk perception by outcomes from the PrEP cascade (n=486).** | | | | | | | | | |
| --- | --- | --- | --- | --- | --- | --- | --- | --- | --- |
|  |  |  | **PrEP status, n (%)** | | | | | |  |
|  | | **Overall Sample** | **Initiation (Baseline)** | **Continued (1-month)** | **Continued  (3-month)** | **Continued  (6-month)** | **Continued consistently to 6-months** | **Any TFV-DP at 3-month** | **Any TFV-DP at 6-month** |
| **Attrition across the PrEP Cascade** | | 486 (100) | 403 (83) | 253 (63) | 212 (54) | 149 (39) | 110 (27) | 85 (49) | 21 (20) |
| **Baseline HIV Risk Score (categorized)** | |  |  |  |  |  |  |  |  |
|  | No/Low HIV risk (score ≤1) | 179 (37) | 147 (82) | 83 (56) | 66 (45) | 47 (34) | 28 (19) | 29 (48) | 5 (14) |
|  | Moderate/High HIV risk (score ≥2) | 307 (63) | 256 (83) | 170 (66) | 146 (59) | 102 (42) | 82 (32) | 56 (49) | 16 (22) |
| **Baseline HIV Risk score, median (IQR)** | | 2 (1-3) | 2 (1-3) | 2 (1-3) | 2 (1-3) | 2 (1-3) | 2 (1-3) | 2 (1-3) | 2 (2-3) |
| **HIV Risk perception at baseline** | |  |  |  |  |  |  |  |  |
|  | No chance | 283 (58) | 237 (84) | 142 (60) | 116 (50) | 83 (37) | 59 (25) | 46 (47) | 5 (8) |
|  | Low chance | 161 (33) | 131 (81) | 87 (66) | 72 (56) | 49 (41) | 36 (27) | 31 (53) | 15 (43) |
|  | High chance | 42 (9) | 35 (83) | 24 (69) | 24 (69) | 17 (50) | 15 (43) | 8 (42) | 1 (10) |
| Abbreviations. TFV-DP=tenofovir disoproxil fumarate/emtricitabine; IQR=interquartile range; PrEP=pre-exposure prophylaxis  Data presented as n(row %) and median (IQR) reported.   Outcome definitions: a) initiation (baseline) are those who initiated PrEP among those PrEP eligible at baseline visit (n=486); b) continuation at 1 are those who attended and requested a PrEP prescription among those who initiated PrEP at baseline (n=403); c) continuation at 3, 6 months are those who attended and requested a PrEP prescription among those who initiated PrEP at baseline and removed those who were censored for pregnancy/infant loss or HIV seroconversion (n=395 for 3-month and n=380 for 6-month); c) continued consistently to 6 months visit are those who attended all study visits (1-,3-, and 6-month follow-up) among those who initiated PrEP at baseline (n=403); d) persisted on PrEP is any TFV-DP detected among those who reported PrEP use in the last 30 days and had dried blood spots analyzed. Those who reported not using PrEP in the last 30 days were marked as “did not adhere” (n=175 at 3 months and n=107 at 6 months). | | | | | | | | | |

| **Supplemental Table 2. Clinical and Behavioral Factors by outcomes from the PrEP cascade (n=486).** | | | | | | | | | |
| --- | --- | --- | --- | --- | --- | --- | --- | --- | --- |
|  |  |  | **PrEP status, n (%)** | | | | | |  |
|  | | **Overall Sample** | **Initiation (Baseline)** | **Continued (1-month)** | **Continued  (3-month)** | **Continued  (6-month)** | **Continued consistently to 6-months** | **Any TFV-DP at 3-month** | **Any TFV-DP at 6-month** |
| **Baseline visit before the national COVID-19 lockdown (March 28, 2020)** | |  |  |  |  |  |  |  |  |
|  | Data collected before | 142 (29) | 131 (92) | 77 (59) | 59 (45) | 34 (27) | 23 (18) | 35 (57) | 8 (26) |
|  | Data collected after | 344 (71) | 272 (79) | 176 (65) | 153 (58) | 115 (45) | 87 (32) | 50 (44) | 13 (17) |
| **AGYW age groups** | |  |  |  |  |  |  |  |  |
|  | Adolescent Girls (Age 16-18) | 77 (16) | 64 (83) | 38 (59) | 39 (62) | 29 (48) | 22 (34) | 17 (55) | 4 (21) |
|  | Young Women (19-24) | 409 (84) | 339 (83) | 215 (63) | 173 (52) | 120 (38) | 88 (26) | 68 (47) | 17 (19) |
| **Maternal age in years at baseline, median (IQR)** | | 21(19-23) | 21(19-23) | 21(19-23) | 21(19-23) | 21(19-23) | 21(19-23) | 21(19-23) | 20(19-22) |
| **Maternal gestational age in weeks at baseline, median (IQR)** | | 24(17-34) | 24(17-33) | 24(17-32) | 24(17-32) | 24(16-34) | 24(16-33) | 25(19-30) | 24(17-35) |
| **Intimate partner violence (IPV) in the past 12 months** | |  |  |  |  |  |  |  |  |
|  | No IPV | 430 (88) | 355 (83) | 220 (62) | 181 (52) | 131 (39) | 94 (26) | 72 (48) | 18 (19) |
|  | IPV reported | 56 (12) | 48 (86) | 33 (69) | 31 (67) | 18 (41) | 16 (33) | 13 (52) | 3 (25) |
| **Baseline STI diagnosis (CT, NG and/or TV)** | |  |  |  |  |  |  |  |  |
|  | No STI | 283 (59) | 235 (83) | 139 (59) | 125 (55) | 85 (39) | 59 (25) | 52 (49) | 12 (21) |
|  | STI Diagnosed | 200 (41) | 166 (83) | 113 (68) | 86 (52) | 64 (41) | 51 (31) | 33 (48) | 9 (18) |
| **Alcohol use in the past 12 months before pregnancy** | |  |  |  |  |  |  |  |  |
|  | No alcohol use | 217 (45) | 180 (83) | 106 (59) | 82 (47) | 59 (35) | 41 (23) | 34 (50) | 8 (18) |
|  | Alcohol use | 269 (55) | 223 (83) | 147 (66) | 130 (59) | 90 (43) | 69 (31) | 51 (48) | 13 (21) |
| **Partner HIV status** | |  |  |  |  |  |  |  |  |
|  | Partner not living with HIV | 319 (66) | 261 (82) | 161 (62) | 131 (51) | 94 (38) | 65 (25) | 47 (44) | 13 (19) |
|  | Don't Know/Partner living with HIV | 167 (34) | 142 (85) | 92 (65) | 81 (59) | 55 (42) | 45 (32) | 38 (56) | 8 (22) |
| **Number of sexual partners** | |  |  |  |  |  |  |  |  |
|  | 1 partner | 392 (81) | 326 (83) | 201 (62) | 166 (52) | 122 (40) | 88 (27) | 67 (48) | 16 (18) |
|  | >1 partners | 94 (19) | 77 (82) | 52 (68) | 46 (61) | 27 (38) | 22 (29) | 18 (51) | 5 (25) |
| **Condom Use** | |  |  |  |  |  |  |  |  |
|  | No | 291 (62) | 238 (82) | 148 (62) | 117 (51) | 83 (37) | 65 (27) | 50 (54) | 15 (24) |
|  | Yes | 178 (38) | 151 (85) | 95 (63) | 85 (56) | 58 (40) | 38 (25) | 29 (41) | 4 (11) |
| **Sexually active at baseline** | |  |  |  |  |  |  |  |  |
|  | Not sexually active | 17 (4) | 14 (82) | 10 (71) | 10 (77) | 8 (62) | 7 (50) | 6 (55) | 2 (25) |
|  | 1-4x per month | 303 (62) | 253 (84) | 154 (61) | 133 (53) | 92 (38) | 66 (26) | 52 (48) | 11 (18) |
|  | >5x per month | 166 (34) | 136 (82) | 89 (65) | 69 (52) | 49 (39) | 37 (27) | 27 (48) | 8 (22) |
| Abbreviations: TFV-DP=tenofovir disoproxil fumarate/emtricitabine; IQR=interquartile range; CT =Chlamydia trachomatis. IPV=intimate partner violence, NG =Neisseria gonorrhoeae, PrEP=pre-exposure prophylaxis, STI=sexually transmitted infection, TV=Trichomonas vaginalis  Data presented as n(row %) and median (IQR) reported.  Outcome definitions: a) initiation (baseline) are those who initiated PrEP among those PrEP eligible at baseline visit (n=486); b) continuation at 1 are those who attended and requested a PrEP prescription among those who initiated PrEP at baseline (n=403); c) continuation at 3, 6 months are those who attended and requested a PrEP prescription among those who initiated PrEP at baseline and removed those who were censored for pregnancy/infant loss or HIV seroconversion (n=395 for 3-month and n=380 for 6-month); c) continued consistently to 6 months visit are those who attended all study visits (1-,3-, and 6-month follow-up) among those who initiated PrEP at baseline (n=403); d) persisted on PrEP is any TFV-DP detected among those who reported PrEP use in the last 30 days and had dried blood spots analyzed. Those who reported not using PrEP in the last 30 days were marked as “did not adhere” (n=175 at 3 months and n=107 at 6 months). | | | | | | | | | |

| **Supplemental Table 3. Clinical and Behavioral Factors associated with outcomes from the PrEP cascade, unadjusted analysis.** | | | | | | | | |
| --- | --- | --- | --- | --- | --- | --- | --- | --- |
|  | | **Initiation (Baseline)** | **Continued (1-month)** | **Continued  (3-month)** | **Continued  (6-month)** | **Continued consistently to 6-months** | **Any TFV-DP at 3-month** | **Any TFV-DP at 6-month** |
| **Baseline Data Collected prior to national COVID-19** | |  |  |  |  |  |  |  |
|  | Yes | Reference |  |  |  |  |  |  |
|  | No, data collected after March 28, 2020 | **0.32 (0.16, 0.62)** | 1.29 (0.84, 1.97) | **1.64 (1.08, 2.51)** | **2.20 (1.38, 3.50)** | **2.21 (1.32, 3.70)** | 0.69 (0.34, 1.43) | 0.84 (0.28, 2.51) |
| **AGYW age groups** | |  |  |  |  |  |  |  |
|  | Adolescent Girls (Age 16-18) | Reference |  |  |  |  |  |  |
|  | Young Women (19-24) | 0.98 (0.51, 1.88) | 1.19 (0.69, 2.05) | 0.67 (0.39, 1.16) | 0.64 (0.37, 1.12) | 0.67 (0.38, 1.18) | 0.84 (0.34, 2.05) | 1.37 (0.37, 5.04) |
| **Maternal age in years at baseline, median (IQR)** | | 1.04 (0.94, 1.15) | 0.98 (0.90, 1.08) | 0.93 (0.85, 1.02) | 0.91 (0.83, 1.00) | 0.92 (0.84, 1.01) | 0.95 (0.82, 1.10) | 0.92 (0.75, 1.13) |
| **Maternal gestational age in weeks at baseline, median (IQR)** | | 1.01 (0.99, 1.04) | 0.99 (0.96, 1.01) | **0.98 (0.96, 1.00)** | 0.99 (0.97, 1.01) | 0.99 (0.96, 1.01) | 1.00 (0.96, 1.04) | 1.04 (0.98, 1.10) |
| **Intimate partner violence (IPV) in the past 12 months** | |  |  |  |  |  |  |  |
|  | No IPV | Reference |  |  |  |  |  |  |
|  | IPV reported | 1.27 (0.58, 2.79) | 1.35 (0.71, 2.58) | **1.92 (1.00, 3.68)** | 1.08 (0.57, 2.05) | 1.39 (0.73, 2.65) | 0.86 (0.34, 2.19) | 0.97 (0.22, 4.35) |
| **Baseline STI diagnosis (CT, NG and/or TV)** | |  |  |  |  |  |  |  |
|  | No STI | Reference |  |  |  |  |  |  |
|  | STI Diagnosed | 1.00 (0.62, 1.62) | 1.47 (0.97, 2.24) | 0.92 (0.61, 1.37) | 1.08 (0.71, 1.64) | 1.32 (0.85, 2.06) | 1.02 (0.50, 2.06) | 0.71 (0.25, 2.06) |
| **Alcohol use in the past 12 months before pregnancy** | |  |  |  |  |  |  |  |
|  | No alcohol use | Reference |  |  |  |  |  |  |
|  | Alcohol use | 1.00 (0.62, 1.60) | 1.35 (0.9, 2.03) | **1.67 (1.12, 2.50)** | 1.39 (0.91, 2.11) | 1.52 (0.97, 2.38) | 1.10 (0.55, 2.22) | 0.94 (0.32, 2.78) |
| **Partner HIV status** | |  |  |  |  |  |  |  |
|  | HIV Negative | Reference |  |  |  |  |  |  |
|  | Don't Know/HIV Positive | 1.26 (0.76, 2.11) | 1.14 (0.75, 1.75) | 1.40 (0.92, 2.13) | 1.17 (0.76, 1.8) | 1.4 (0.89, 2.2) | 1.67 (0.81, 3.42) | 1.07 (0.36, 3.16) |
| **Number of sexual partners** | |  |  |  |  |  |  |  |
|  | 1 partner | Reference |  |  |  |  |  |  |
|  | >1 partners | 0.92 (0.51, 1.65) | 1.29 (0.76, 2.19) | 1.41 (0.85, 2.35) | 0.92 (0.54, 1.55) | 1.08 (0.62, 1.88) | 1.28 (0.53, 3.12) | 1.52 (0.42, 5.53) |
| **Condom Use** | |  |  |  |  |  |  |  |
|  | No | Reference |  |  |  |  |  |  |
|  | Yes | 1.25 (0.75, 2.07) | 1.03 (0.68, 1.57) | 1.26 (0.83, 1.90) | 1.14 (0.74, 1.75) | 0.9 (0.56, 1.43) | 0.60 (0.29, 1.25) | 0.35 (0.10, 1.23) |
| **Sexually active at baseline** | |  |  |  |  |  |  |  |
|  | Not sexually active | Reference |  |  |  |  |  |  |
|  | 1-4x per month | 1.08 (0.30, 3.91) | 0.62 (0.19, 2.04) | 0.34 (0.09, 1.27) | 0.39 (0.12, 1.22) | 0.35 (0.12, 1.05) | 0.77 (0.18, 3.27) | 0.44 (0.06, 3.54) |
|  | >5x per month | 0.97 (0.26, 3.59) | 0.76 (0.23, 2.55) | 0.33 (0.09, 1.25) | 0.39 (0.12, 1.27) | 0.37 (0.12, 1.14) | 0.90 (0.20, 4.13) | 0.57 (0.07, 4.88) |
| Abbreviations: TFV-DP=tenofovir disoproxil fumarate/emtricitabine; IQR=interquartile range; CT =Chlamydia trachomatis. IPV=intimate partner violence, NG =Neisseria gonorrhoeae, PrEP=pre-exposure prophylaxis, STI=sexually transmitted infection, TV=Trichomonas vaginalis  Bold: statistically significant measures that do not cross the null (1.00). N(%) for this table can be found in Supplemental Table 2. NE= Not estimated due to insufficient sample size for a logistic regression.  Outcome definitions: a) initiation (baseline) are those who initiated PrEP among those PrEP eligible at baseline visit (n=486); b) continuation at 1 are those who attended and requested a PrEP prescription among those who initiated PrEP at baseline (n=403); c) continuation at 3, 6 months are those who attended and requested a PrEP prescription among those who initiated PrEP at baseline and removed those who were censored for pregnancy/infant loss or HIV seroconversion (n=395 for 3-month and n=380 for 6-month); c) continued consistently to 6 months visit are those who attended all study visits (1-,3-, and 6-month follow-up) among those who initiated PrEP at baseline (n=403); d) persisted on PrEP is any TFV-DP detected among those who reported PrEP use in the last 30 days and had dried blood spots analyzed. Those who reported not using PrEP in the last 30 days were marked as “did not adhere” (n=175 at 3 months and n=107 at 6 months). | | | | | | | | |

| **Supplemental Table 4 Clinical and Behavioral Factors associated with outcomes from the PrEP cascade, adjusted analysis.** | | | | | | | | |
| --- | --- | --- | --- | --- | --- | --- | --- | --- |
|  | | **Initiation (Baseline)** | **Continued (1-month)** | **Continued  (3-month)** | **Continued  (6-month)** | **Continued consistently to 6-months** | **Any TFV-DP at 3-month** | **Any TFV-DP at 6-month** |
| **Baseline Data Collected prior to national COVID-19** | |  |  |  |  |  |  |  |
|  | Yes | Reference |  |  |  |  |  |  |
|  | No, data collected after March 28, 2020 | **0.31 (0.16, 0.61)** | 1.31 (0.85, 2.02) | **1.68 (1.09, 2.58)** | **2.19 (1.37, 3.51)** | **2.21 (1.31, 3.73)** | 0.59 (0.22, 1.62) | 0.54 (0.19, 1.53) |
| **AGYW age groups** | |  |  |  |  |  |  |  |
|  | Adolescent Girls (Age 16-18) | Reference |  |  |  |  |  |  |
|  | Young Women (19-24) | 0.59 (0.23, 1.52) | 1.73 (0.77, 3.91) | 0.89 (0.40, 1.99) | 0.98 (0.43, 2.24) | 1.00 (0.42, 2.37) | 0.90 (0.26, 3.05) | NE |
| **Maternal age in years at baseline, median (IQR)** | | 1.04 (0.94, 1.16) | 0.98 (0.89, 1.07) | 0.92 (0.84, 1.01) | **0.91 (0.83, 1.00)** | 0.92 (0.83, 1.02) | 0.94 (0.81, 1.09) | 0.88 (0.72, 1.07) |
| **Maternal gestational age in weeks at baseline, median (IQR)** | | 1.02 (0.99, 1.04) | 0.98 (0.96, 1.01) | **0.97 (0.95, 1.00)** | **0.98 (0.96, 1.00)** | **0.98 (0.96, 1.00)** | 1.00 (0.96, 1.04) | 1.02 (0.97, 1.07) |
| **Intimate partner violence (IPV) in the past 12 months** | |  |  |  |  |  |  |  |
|  | No IPV | Reference |  |  |  |  |  |  |
|  | IPV reported | 1.31 (0.59, 2.91) | 1.33 (0.69, 2.55) | **1.96 (1.01, 3.80)** | 1.12 (0.58, 2.16) | 1.38 (0.71, 2.67) | 1.43 (0.35, 5.81) | 1.36 (0.30, 6.11) |
| **Baseline STI diagnosis (CT, NG and/or TV)** | |  |  |  |  |  |  |  |
|  | No STI | Reference |  |  |  |  |  |  |
|  | STI Diagnosed | 0.98 (0.60, 1.62) | 1.46 (0.96, 2.24) | 0.85 (0.56, 1.28) | 1.02 (0.66, 1.57) | 1.28 (0.81, 2.03) | 0.82 (0.31, 2.16) | 0.76 (0.28, 2.07) |
| **Alcohol use in the past 12 months before pregnancy** | |  |  |  |  |  |  |  |
|  | No alcohol use | Reference |  |  |  |  |  |  |
|  | Alcohol use | 1.02 (0.63, 1.65) | 1.32 (0.88, 1.99) | **1.65 (1.10, 2.49)** | 1.41 (0.92, 2.17) | 1.51 (0.96, 2.40) | 1.23 (0.46, 3.27) | 1.16 (0.41, 3.29) |
| **Partner HIV status** | |  |  |  |  |  |  |  |
|  | HIV Negative | Reference |  |  |  |  |  |  |
|  | Don't Know/HIV Positive | 1.32 (0.78, 2.22) | 1.15 (0.75, 1.76) | 1.41 (0.92, 2.17) | 1.17 (0.75, 1.82) | 1.40 (0.88, 2.21) | 1.21 (0.45, 3.25) | 1.25 (0.46, 3.44) |
| **Number of sexual partners** | |  |  |  |  |  |  |  |
|  | 1 partner | Reference |  |  |  |  |  |  |
|  | >1 partners | 0.90 (0.49, 1.64) | 1.29 (0.76, 2.19) | 1.41 (0.84, 2.37) | 0.94 (0.55, 1.62) | 1.10 (0.63, 1.94) | 1.48 (0.47, 4.66) | 1.64 (0.50, 5.33) |
| **Condom Use** | |  |  |  |  |  |  |  |
|  | No | Reference |  |  |  |  |  |  |
|  | Yes | 1.17 (0.70, 1.96) | 1.05 (0.68, 1.61) | 1.28 (0.84, 1.96) | 1.18 (0.76, 1.84) | 0.94 (0.58, 1.52) | 0.38 (0.12, 1.25) | 0.30 (0.08, 1.10) |
| **Sexually active at baseline** | |  |  |  |  |  |  |  |
|  | Not sexually active | Reference |  |  |  |  |  |  |
|  | 1-4x per month | 0.99 (0.27, 3.66) | 0.62 (0.19, 2.05) | 0.34 (0.09, 1.3) | 0.40 (0.12, 1.31) | 0.37 (0.12, 1.12) | 0.65 (0.16, 3.64) | 0.70 (0.12, 4.07) |
|  | >5x per month | 0.87 (0.23, 3.33) | 0.75 (0.22, 2.53) | 0.33 (0.08, 1.28) | 0.42 (0.13, 1.43) | 0.39 (0.12, 1.24) | 0.83 (0.14, 4.91) | 0.94 (1.53, 5.77) |
| Abbreviations: TFV-DP=tenofovir disoproxil fumarate/emtricitabine; IQR=interquartile range; CT =Chlamydia trachomatis. IPV=intimate partner violence, NG =Neisseria gonorrhoeae, PrEP=pre-exposure prophylaxis, STI=sexually transmitted infection, TV=Trichomonas vaginalis  Bold: statistically significant measures that do not cross the null (1.00). N(%) for this table can be found in Supplemental Table 2. NE= Not estimated due to insufficient sample size for a logistic regression.  Outcome definitions: a) initiation (baseline) are those who initiated PrEP among those PrEP eligible at baseline visit (n=486); b) continuation at 1 are those who attended and requested a PrEP prescription among those who initiated PrEP at baseline (n=403); c) continuation at 3, 6 months are those who attended and requested a PrEP prescription among those who initiated PrEP at baseline and removed those who were censored for pregnancy/infant loss or HIV seroconversion (n=395 for 3-month and n=380 for 6-month); c) continued consistently to 6 months visit are those who attended all study visits (1-,3-, and 6-month follow-up) among those who initiated PrEP at baseline (n=403); d) persisted on PrEP is any TFV-DP detected among those who reported PrEP use in the last 30 days and had dried blood spots analyzed. Those who reported not using PrEP in the last 30 days were marked as “did not adhere” (n=175 at 3 months and n=107 at 6 months). | | | | | | | | |

Supplemental Figure 1. HIV PrEP Cascade among Pregnant and Postpartum Women by Age Categories.


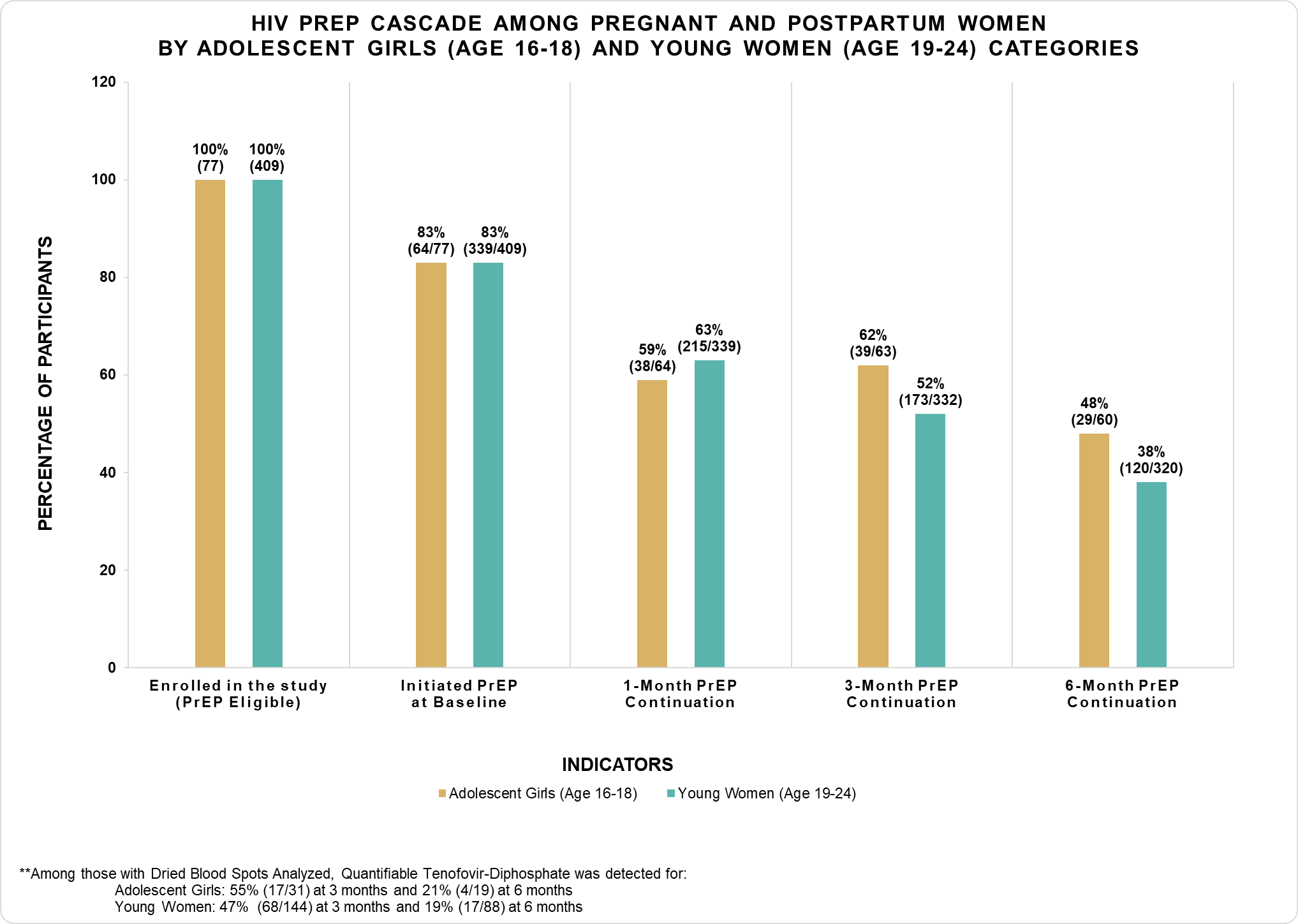


Supplemental Figure 2. HIV PrEP Cascade among Pregnant and Postpartum by HIV Risk Score Categories.

**
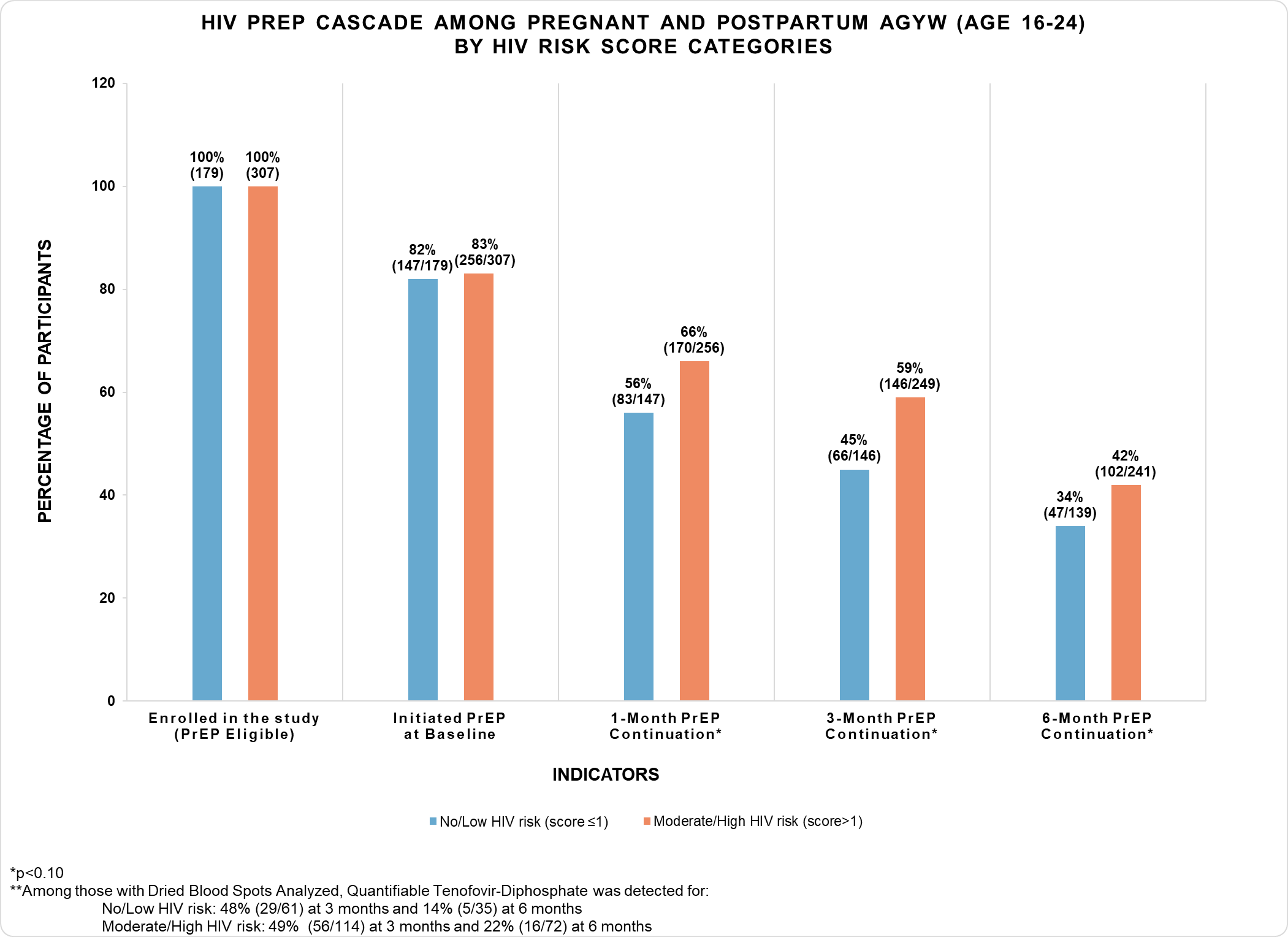
**
